# Accurate and Efficient Spin–Phonon Coupling and Spin Dynamics Calculations for Molecular Solids

**DOI:** 10.1021/jacs.3c06015

**Published:** 2023-11-02

**Authors:** Rizwan Nabi, Jakob K. Staab, Andrea Mattioni, Jon G. C. Kragskow, Daniel Reta, Jonathan M. Skelton, Nicholas F. Chilton

**Affiliations:** † Department of Chemistry, University of Manchester, Manchester M13 9PL, U.K.; ‡ Department of Chemistry, University of Bath, Bath BA2 7AY, U.K.; § Faculty of Chemistry, University of the Basque Country UPV/EHU, 20018 Donostia, Spain; ∥ Donostia International Physics Center (DIPC), 20018 Donostia, Spain; ⊥ IKERBASQUE, Basque Foundation for Science, 48013 Bilbao, Spain

## Abstract

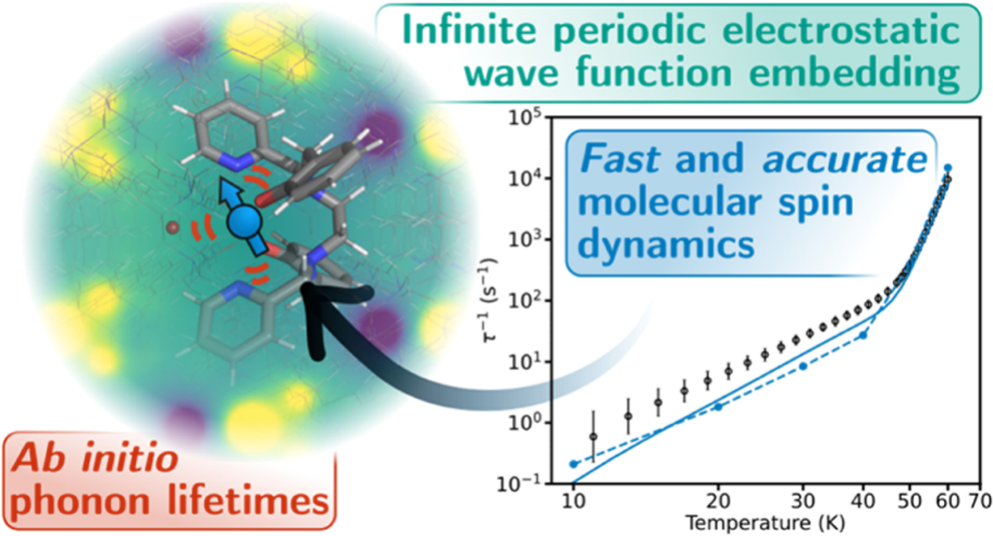

Molecular materials are poised to play a significant role in the development of future optoelectronic and quantum technologies. A crucial aspect of these areas is the role of spin–phonon coupling and how it facilitates energy transfer processes such as intersystem crossing, quantum decoherence, and magnetic relaxation. Thus, it is of significant interest to be able to accurately calculate the molecular spin–phonon coupling and spin dynamics in the condensed phase. Here, we demonstrate the maturity of *ab initio* methods for calculating spin–phonon coupling by performing a case study on a single-molecule magnet and showing quantitative agreement with the experiment, allowing us to explore the underlying origins of its spin dynamics. This feat is achieved by leveraging our recent developments in analytic spin–phonon coupling calculations in conjunction with a new method for including the infinite electrostatic potential in the calculations. Furthermore, we make the first *ab initio* determination of phonon lifetimes and line widths for a molecular magnet to prove that the commonplace Born–Markov assumption for the spin dynamics is valid, but such “exact” phonon line widths are not essential to obtain accurate magnetic relaxation rates. Calculations using this approach are facilitated by the open-source packages we have developed, enabling cost-effective and accurate spin–phonon coupling calculations on molecular solids.

## Introduction

Spin–phonon coupling and molecular spin dynamics govern crucial processes in molecule-based imaging, optoelectronics, and quantum technologies.^1^ These include contrast magnetic resonance imaging in healthcare,^2^ singlet fission for energy harvesting,^3^ ultrafast excited-state dynamics for energy transfer,^4,5^ long-lived optical states for optical quantum interfaces,^6^ and decoherence processes in qubits.^7^ Spin–phonon coupling also determines the performance of single-molecule magnets (SMMs), which are convenient platforms for probing fundamental molecular spin physics.^5,8−10^ SMMs are molecules that possess magnetic bistability and show magnetic memory effects at low temperature in the absence of long-range order.^11,12^ The time scale for magnetic memory, τ, is set by the interactions of a molecule with its environment and is mediated by phonons in the solid state.^13,14^ The same interactions limit the time scale of quantum coherence when other mechanisms are suppressed.^7^ For both SMMs and molecular qubits, the goal is to slow the spin dynamics to ensure that magnetic memory and quantum phase coherence remain for as long as possible.

Loss of magnetic memory in SMMs, also known as magnetic reversal or magnetic relaxation, occurs through different processes dictated by spin dynamics. Single-phonon interactions lead to magnetic relaxation *via* the Orbach mechanism, for which the characteristic time has an exponential temperature dependence τ = τ_0_ exp(*U*_eff_/*k*_B_*T*) with a characteristic phonon time scale τ_0_ and an energy barrier *U*_eff_. In the best-performing SMMs, this is driven by high-energy optic phonons.^15,16^ At lower temperatures, the populations of high-energy phonons are very low, and the Orbach mechanism is suppressed. In this regime, the spin dynamics are instead dominated by two-phonon Raman mechanisms driven by low-energy (pseudo)acoustic phonons, showing a power law temperature dependence of τ in the range of *T*^–1^–*T*^–7^.^17^ Other mechanisms pertinent to the spin dynamics of SMMs, such as the direct and quantum tunneling of magnetization (QTM) mechanisms,^11^ are not discussed here as they are either not relevant in zero magnetic field (direct) or are only active at very low temperatures (QTM; usually <10 K).

Among the current best-performing SMMs, magnetic hysteresis has been observed as high as 80 K for [Dy(Cp*)(Cp^*i*^Pr_5_)][B(C_6_F_5_)_4_] (Cp* = pentamethyl cyclopentadienyl)^18^ and more recently for the mixed-valence Cp^iPr5^DyI_3_DyCp^iPr5^ (Cp^iPr5^ = pentaisopropyl cyclopentadienyl), which shows strong magnetic exchange coupling mediated by the Ln–Ln half-σ bond.^19^ In both cases, a large axial magnetic anisotropy is imposed by the cyclopentadienyl ligands, leading to large *U*_eff_ values and slow Orbach relaxation. For [Dy(Cp^ttt^)_2_][B(C_6_F_5_)_4_] (Cp^ttt^ = C_5_H_2_-1,2,4-^*t*^Bu),^15^ it has been demonstrated that the comparatively slow spin dynamics in the Raman regime for this class of materials results from a separation in energy between the very high-energy optical phonons (due to the conjugated five-membered rings being the only ligands in the first coordination sphere) and low-energy pseudoacoustic phonons (due to the soft intermolecular potential energy of the bulky cation–anion pair).^20^ Variation of the cyclopentadienyl substituents can have a significant effect on the spin dynamics^15,18,21^ since it impacts both the magnetic anisotropy and the vibrational spectrum.^22^ Despite these successes, control of the spin dynamics through chemical modification remains an open challenge for the design of improved SMMs and indeed any molecular spin system, and given the vastness of chemical space, strategies employing machine learning are likely to be particularly promising.^23^

**Figure 1:**
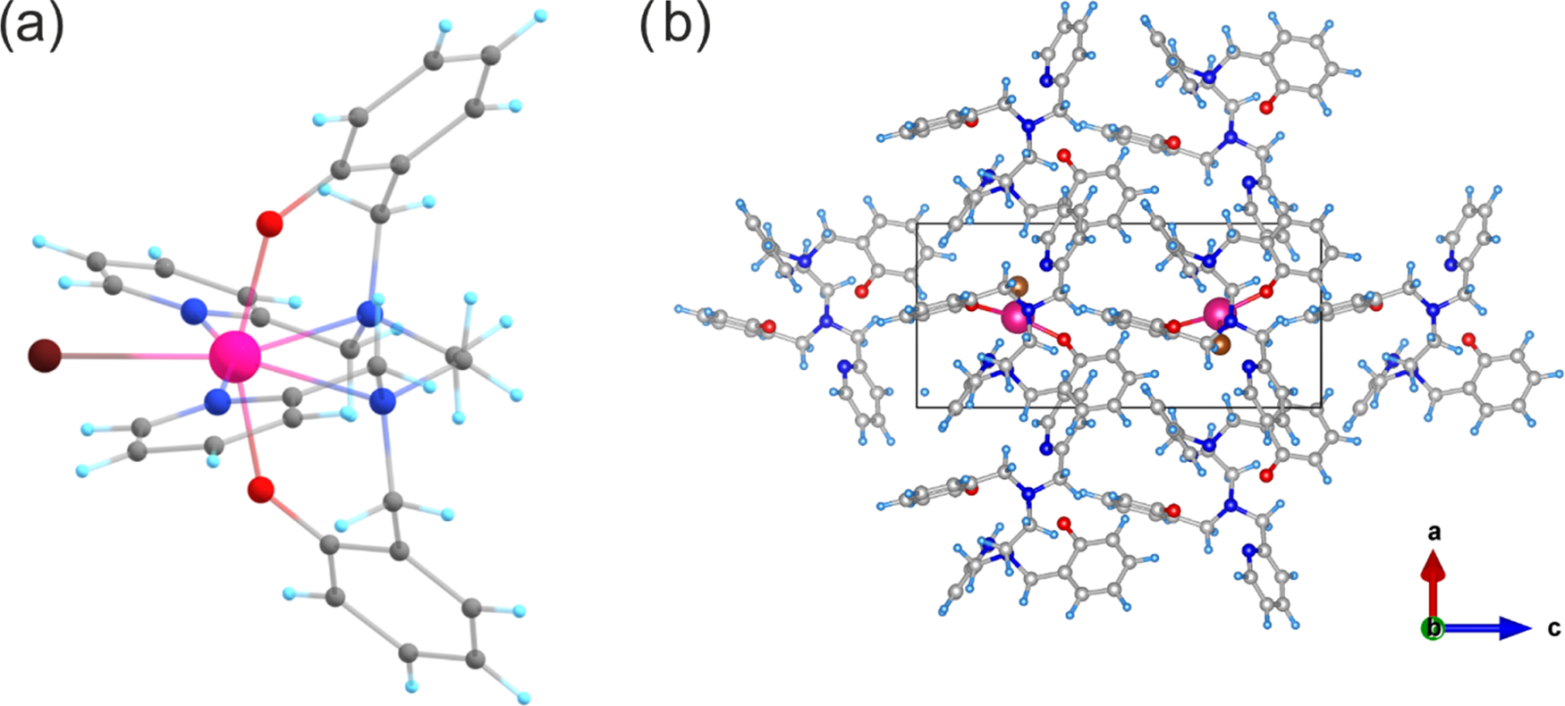
(a) Molecular structure of **1** from X-ray diffraction.^32^ (b) Optimized conventional unit cell containing two molecules of **1**. The atoms are color-coded as follows: Dy = pink, N = dark blue, Br = brown, O = red, C = gray, and H = light blue.

To this end, a number of research groups have recently started to develop *ab initio* methods for calculating molecular spin–phonon coupling and modeling spin dynamics.^22,24−27^ Such methods are crucial to leverage the computational material design procedures^28^ that have proven very successful in solid-state chemistry^29,30^ to discover new and improved molecular materials with desirable spin dynamics. However, for this to be a viable approach, the calculations must approach experimental accuracy. Just recently, Lunghi and co-workers presented a landmark study: the first fully *ab initio* simulation of Raman spin dynamics for an SMM.^31^ Taking inspiration from their work, we herein introduce a number of further developments, resulting in the most accurate *ab initio* simulation of molecular spin dynamics to date. We focus on the high-performance Dy(III) SMM, [Dy(bbpen)Br] (**1**, Figure 1),^32^ chosen because of its small unit cell size, relatively high-symmetry space group (*C*222_1_), and excellent SMM performance, although we note that the methodology described herein is equally applicable to any other molecular magnet, and numerous other such preliminary studies are underway in our group. We perform the first *ab initio* calculation of the phonon line widths for a molecular magnet to show that: i)phonon lifetimes are orders of magnitude shorter than the spin lifetimes in this compound, justifying the commonly assumed Born–Markov approximation for molecular spin dynamics,^22,31^ and ii) the phonon line widths are highly energy- and wavevector-dependent, with full width at half maximum (fwhm) values varying between 0.1 and 40 cm^–1^ at 300 K. We find that the spin dynamics are relatively insensitive to the choice of line width model, with similar results obtained using a fixed line width or a thermodynamic approximation,^25^ and thus our work suggests that the burden of performing expensive phonon line width calculations for other molecules may not be required for accurate spin dynamics simulations. Furthermore, we show that the use of finite slab methods leads to significant errors and that accurate treatment of the electrostatic (Madelung) potential of the infinite crystal lattice is essential to achieve quantitative accuracy. Our open-source tools implement a computationally inexpensive and accurate method to calculate the infinite crystalline electrostatic potential, including its effect on the spin–phonon coupling terms, which vastly improves the agreement with the experiment. This work thus paves the way for accurate and efficient spin–phonon calculations on solid-state molecular systems with applicability beyond molecular magnetism.

## Methods

A brief overview of the entire method is as follows: (1) geometry optimization (either a single molecule of **1** in the gas phase or an infinite perfectly periodic crystal of **1**); (2) calculation of the vibrations (gas phase) or phonons (crystalline phase); (3) calculation of the electronic structure of a single molecule of **1** (either on its own in the gas phase or embedded in an electrostatic representation of the crystal); (4) calculation of the spin–phonon coupling between the vibrational/phonon modes and the electronic states in **1** (again, either on its own in the gas phase or embedded in an electrostatic representation of a crystal); and (5) calculation of the magnetic relaxation rates.

Geometry optimization of a single neutral molecule of **1** in the gas phase was performed with Gaussian09d^33^ using the PBE functional and the D3 semiempirical dispersion correction.^34,35^ Density functional theory (DFT) is based on a single configuration wave function and therefore struggles with the multiconfigurational ground state arising from near-degenerate 4f electronic configurations. To avoid this, we substituted Dy(III) for the chemically and structurally analogous Y(III), which has no 4f electrons, and used the Stuttgart RSC 1997 effective core potential (ECP) and associated valence basis set for Y and the cc-pVDZ basis sets for all other atoms.^36^ We found a root-mean-square deviation (RMSD) for all the atomic positions compared to the experimental crystal structure of 0.13 Å. We then calculated the vibrational modes of the gas-phase molecule of **1** using analytic methods in Gaussian09d, where the isotopic mass of Y was set as the isotopic mass of Dy.

Geometry optimization of the crystalline phase of **1** was performed using the primitive cell, with starting atomic positions and unit cell parameters obtained from the Cambridge Structural Database (CCDC: 1416543), using periodic density functional theory (DFT) as implemented in VASP 5.4.4.^37−40^ The PBE functional^34^ with the D3 semiempirical dispersion correction^35^ was employed to model the electron exchange and correlation. To avoid the multiconfigurational ground state of Dy(III) in this case, we used a 4f-in-core ECP for Dy(III); we have previously verified that both Y(III) substitution and a 4f-in-core ECP give the same results. All ion cores were modeled with projector augmented wave (PAW) pseudopotentials,^41,42^ and the valence electronic structure was modeled using a plane-wave basis set with an energy cutoff of 800 eV and Γ-point Brillouin zone sampling, with both parameters determined *via* explicit convergence testing. Starting from the 128-atom primitive cell of the published X-ray structure of **1**, the atomic positions and unit cell parameters were optimized to tight tolerances of 10^–8^ eV on the electronic total energy and 10^–2^ eV Å^–1^ on the forces. Phonon calculations were then performed with the optimized crystal structure of **1** using the finite-difference method implemented in Phonopy^43^ and Phono3py.^44^ In this approach, the Phonopy code is used to generate a series of distorted structures (with each independent atom shifted along each independent *xyz* coordinate, one by one, by 0.01 Å) and the forces on the atoms in each distorted structure are calculated using VASP, where the outputs are combined with Phonopy again to obtain the Hessian matrix of second derivatives of the energy with respect to coordinates. The Hessian is then “mass-weighted” using the atomic masses to give the dynamical matrix, which is diagonalized to give the normal modes of vibration and squared frequencies. The process is slightly more involved than this, as the calculations are periodic, and we refer the interested reader to our recent Tutorial Review, which covers this in detail.^45^ The Phono3py code is similarly used to generate a sequence of distorted structures in which pairs of atoms are displaced to obtain the third-order force constants, which are combined with the harmonic frequencies and eigenvectors to determine the phonon lifetimes and line widths. A 2 × 2 × 1 supercell with 512 atoms was employed to determine the second-order force constants in Phonopy,^43^ while the third-order force constants were determined for the primitive cell (i.e., a 1 × 1 × 1 cell) using Phono3py,^44^ and the phonon frequencies and line widths were evaluated on uniform 2 × 2 × 2, 3 × 3 × 3, 4 × 4 × 4, and 5 × 5 × 5 *q*-point grids using Fourier interpolation with Phono3py.

We have adapted our established protocol for modeling molecular spin dynamics^22^ to take into account the phonon modes in the crystalline phase. We first take each unique molecule in the optimized unit cell (expanded to contain complete molecules) and perform a gas-phase DFT calculation using Gaussian 09d^33^ with the PBE functional^34^ to determine the atomic charges required to reproduce the external molecular electrostatic potential using the CHELPG method.^46^ To avoid the multiconfigurational ground state inherent to Dy(III) in these molecular DFT calculations, we substituted Dy(III) for the chemically and structurally analogous Y(III) and use the Stuttgart RSC 1997 effective core potential (ECP) and associated valence basis set for Y,^47−49^ along with the cc-pVDZ basis sets for all other atoms.^36^

To calculate the spin–phonon coupling, we must consider how the vibrational/phononic (i.e., structural) degrees of freedom influence the electronic structure of the Dy(III)-based SMM. Determination of the electronic structure of a molecule with a multiconfigurational ground state requires explicitly correlated electronic structure methods. Our method of choice is complete active space self-consistent field (CASSCF) theory, which is efficient and accurate for describing the magnetic properties of Ln(III) SMMs.^50^ There is no problem using this method for our single-molecule gas-phase structural and vibrational model for **1**. However, for adapting our method to the crystalline phase, we note that the CASSCF is not compatible with periodic wave functions, and hence, we must bridge the gap between the phonons described in the infinite periodic crystal and the electronic structure described with a finite model. The simplest approach is a finite slab of unit cells cropped from the infinite periodic crystal (approach 1), but we find that this has its shortcomings, and hence, we have implemented a more accurate method accounting for the infinite crystalline electrostatic potential (approach 2); both methods are implemented in our *spin_phonon_suite* code (version 1.4.1).^51^

### Approach 1: Finite Slab Expansions

The set of phonon wavevectors *q* on a Γ-centered *q*-point sampling grid with *q*_1_ × *q*_2_ × *q*_3_ subdivisions is commensurate with a *q*_1_ × *q*_2_ × *q*_3_ finite slab cell expansion in the real space (i.e., the slab is of the correct size to contain an integer number of phonon wavelengths for all the *q* on the sampling grid). For a given *q*-point grid, we build the required *q*_1_ × *q*_2_ × *q*_3_ finite slab, redefined by translation to position a single molecule of **1** in the center. The electrostatic potential of the finite slab is accounted for by assigning the remaining atoms (excluding the central molecule) their CHELPG charges determined as outlined above.

### Approach 2: Infinite Crystalline Electrostatic Potential *via* Conductor Screening

In this method, we build a finite array of unit cells with an approximately spherical shape of approximately ∼40 Å radius, chosen by convergence testing (Figure S1), with the unit cell redefined by translation to position a single molecule of **1** in the center of the spherical array. All remaining atoms in the spherical array are assigned their CHELPG charges (outlined above), and the entire array is embedded in a perfect conductor reaction field cavity with a radius of 40 Å using the Kirkwood solvent model with ε = ∞.^52^ The displacement vectors for phonons corresponding to arbitrary *q*_1_ × *q*_2_ × *q*_3_ grids can then be mapped onto the spherical array as required.

In both approaches and for our gas-phase calculations, the electronic structure of the central molecule is obtained with a state-average CASSCF spin–orbit (SA-CASSCF-SO) calculation in OpenMolcas 23.02 (modified to allow up to 50,000 atoms).^53^ Here, we consider 18 *S* = 5/2 states (^6^H and ^6^F terms) for a 9-in-7 active space (4f^9^ configuration) using the second-order Douglas–Kroll–Hess relativistic decoupling,^54^ the Cholesky “atomic compact” resolution of the identity method for approximating the two-electron integrals,^55^ and ANO-RCC basis sets for all atoms (VTZP for Dy, VDZP for the first coordination sphere, and VDZ for all other atoms).^56,57^ These 18 spin-free states are then mixed with SO coupling, and the lowest 16 resulting states (the ^6^H_15/2_ multiplet) are projected onto a crystal field (CF) Hamiltonian acting in the (2*J* + 1)-dimensional |*m*_*J*_⟩ basis, using our *angmom_suite* code (version 1.17.1).^58^ The spin–phonon coupling parameters for each phonon mode, defined by band index *j* and wavevector *q*, are the derivatives of the CF parameters along the phonon normal mode vectors.^22,26,45,59^ To compute these, we first obtain the derivatives of the CF parameters with respect to Cartesian atomic coordinates using an analytic linear vibronic coupling (LVC) model^60^ and then convert to the normal mode basis using the linear combination of atomic displacements specified by the mode displacement vector.^45^ This is done for all vibrational modes of the gas-phase model and all phonon modes at all *q*-points in the sampling mesh for the crystalline-phase model.

**Figure 2:**
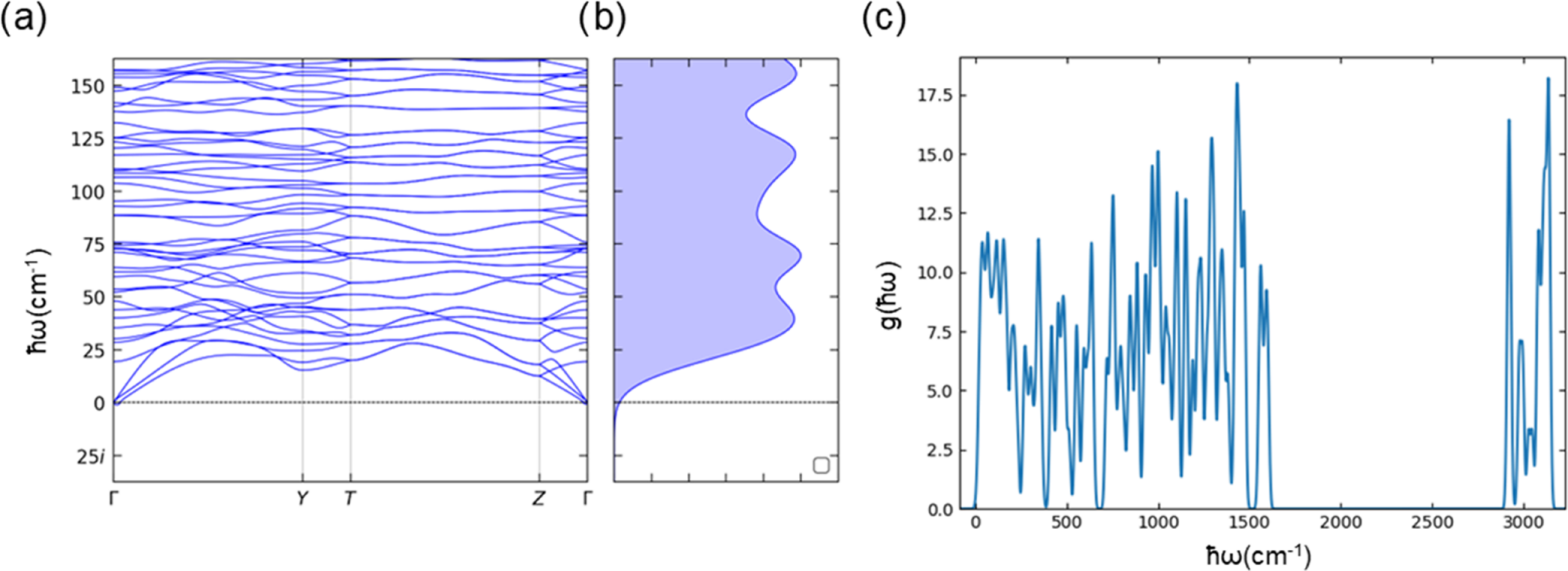
Calculated phonon spectrum for the crystal structure of **1**. (a, b) Low-energy dispersion and phonon density of states (DoS). (c) DoS over the full energy range from 0 to 3500 cm^–1^. Both DoS plots are calculated for an 8 × 8 × 8 *q*-point grid using Gaussian lineshapes with a fwhm of 17.5 cm^–1^.

Magnetic relaxation rates are then determined using our *Tau* code (commit e058b24959),^61^ considering Orbach and Raman rates,^26,45,62^ given by eqs 40, 41, and 46–49 in reference.^45^ There are two forms of the Raman mechanism, which arise from their derivation using different orders of perturbation theory:^26,45,62^ the Raman-I mechanism (first-order in spin–phonon coupling, second-order in time) does not depend on the magnitude of an external magnetic field,^63^ while the Raman-II mechanism (second-order in spin–phonon coupling, first-order in time) has a quadratic dependence on the field and vanishes in zero field.^20^ Since our experiments are performed in zero field, we do not consider the Raman-II mechanism, and we therefore refer to the Raman-I mechanism simply as “the Raman mechanism” throughout. In the context of magnetic relaxation in SMMs, the two-phonon Raman mechanism concerns coupling between the two states of the ground Kramers doublet, and our calculations are thus restricted to this pair of states. Derivation of the Raman rate expressions adopts the secular approximation, which assumes that no degeneracies exist in the electronic eigenstates,^26,31,45^ and thus, we must introduce an energy gap between the two states of the ground Kramers doublet. Indeed, this occurs in experiments due to the presence of a dipolar magnetic field and/or the driving AC magnetic field, and we therefore apply a magnetic field of 2 Oe along the main magnetic axis of the molecule, splitting the ground doublet by ca. 0.002 cm^–1^. The Raman mechanism involves pairs of phonons, and the rate is obtained as the double integral over their lineshapes.^26,45,60^ We restrict the domain of the phonon energies to 0 ≤ ω ≲ ω_cut_, where ω_cut_ = 267 cm^–1^ is chosen as the minimum in the phonon density of states (DoS) above the low-energy pseudoacoustic peak (Figure 2c). The cutoff is applied to avoid divergences in the Raman rates, as the denominator of eqs 46–49 in ref (45) goes to zero when ℏω is resonant with a CF excitation, and the chosen value is sufficiently smaller than the first crystal field excitation of ca. 420 cm^–1^. The double integral is transformed into a one-dimensional integral due to the conservation of energy *via* the Dirac delta function and is performed over anti-Lorentzian phonon lineshapes (eq 11 in ref (45)) to an equivalent range of μ ± 2σ (95%) using the trapezoidal method with 40 equidistant steps (anti-Lorentzian lineshapes are used to ensure that the DoS goes to zero at zero energy).

## Results and Discussion

### Phonons and Phonon Line Widths

Compound **1** is a monometallic Dy(III) molecule with a pentagonal bipyramidal coordination geometry. The molecule crystallizes in the orthorhombic space group *C*222_1_ with half a molecule in the asymmetric unit and two complete molecules in the conventional unit cell (Figure 1).^32^ To obtain the phonon spectrum of **1**, we first optimize the unit cell parameters and atomic positions of **1** with periodic DFT (see the Methods), starting from the experimental crystal structure, and observe only very small changes: the optimized cell parameters are very similar to the measured values (Table S1), and we find an RMSD for all atomic positions of only 0.10 Å. This indicates that our chosen methodology is a good approximation to the molecular forces. We then obtain the second derivatives of the energy (first derivative of the forces) with respect to the atomic positions using numerical finite differences and construct the dynamical matrix to determine the normal modes of vibration by matrix diagonalization (see the Methods).^45^

**Figure 3:**
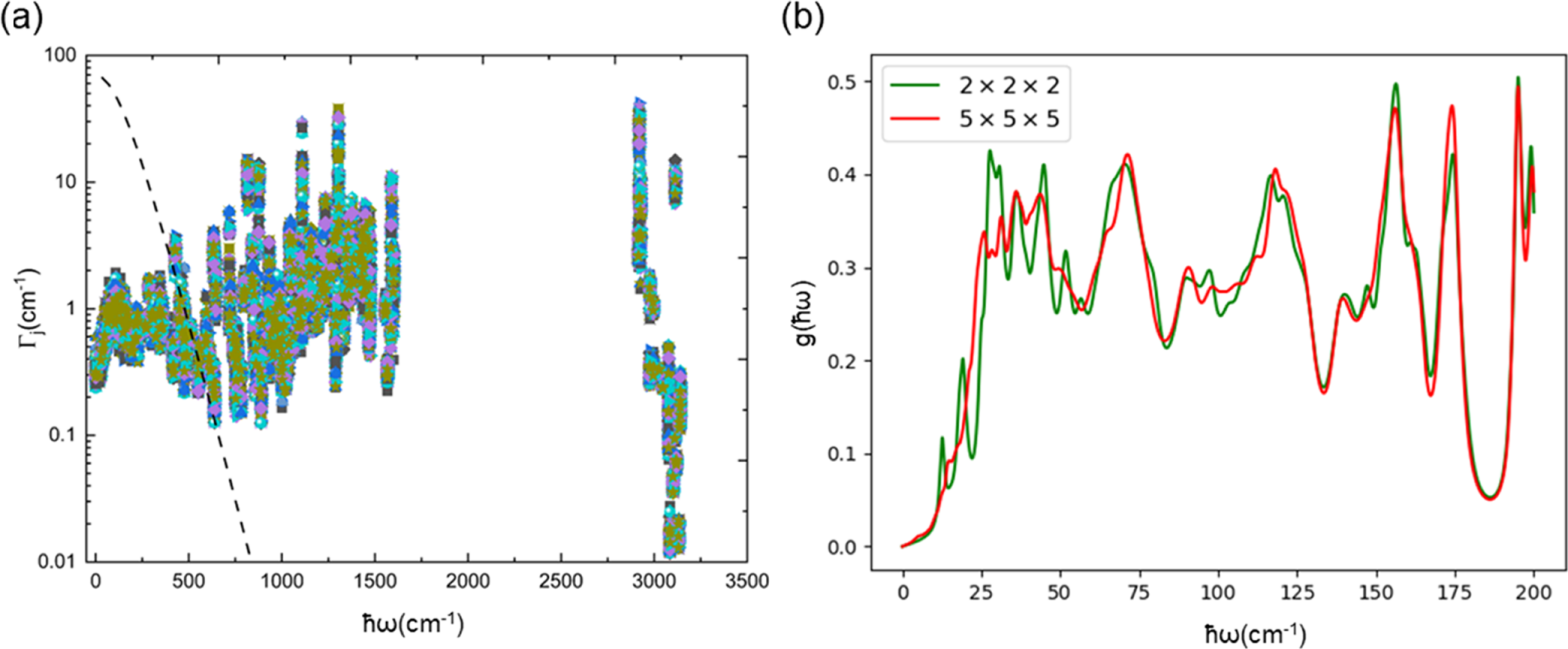
(a) Phonon line widths as a function of mode energy at 50 K for the 27 unique *q*-points on a 5 × 5 × 5 Brillouin zone sampling mesh. The *q*-points are distinguished by different colors and markers. The dashed line shows the predicted line width as a function of mode energy obtained using eq 1. (b) Low-energy DoS at 50 K constructed from the *ab initio* frequencies and line widths obtained on 2 × 2 × 2 and 5 × 5 × 5 *q*-point grids using an anti-Lorentzian line shape.

The low-energy phonon dispersion (Figure 2a) comprises the three acoustic modes, corresponding to rigid translations with zero energy at the Γ-point, and a high density of dispersive pseudoacoustic modes that arise predominantly from combinations of rigid molecular translations and rotations. The DoS shows a high-density continuum of phonon modes below ca. 250 cm^–1^ (Figure 2b), while the complete spectrum extends up to 3500 cm^–1^ and at higher frequencies comprises relatively flat bands of intramolecular modes (Figure 2c). Phonons have finite lifetimes τ_*qj*_ due to a variety of scattering processes, which means that the intrinsic line widths Γ_*qj*_ = ℏ/τ_*qj*_ (where Γ_*qj*_ is the Lorentzian fwhm line width) vary as a function of energy and wavevector and are intrinsically temperature-dependent through the populations of the modes involved in the scattering processes.^44^ We have previously treated phonon line widths as an empirical parameter,^22^ while Lunghi et al. proposed a simplified model for an effective phonon line width based on the NVT canonical ensemble (eq 1)^25^
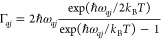
1To obtain the lifetimes τ_*qj*_ and line widths Γ_*qj*_ from first principles, we calculated third-order (anharmonic) force constants with a similar numerical finite-difference method (see the Methods) and modeled the phonon–phonon scattering processes explicitly for a grid of wavevectors *q* at a series of temperatures.^44^ Here, we have used 2 × 2 × 2, 3 × 3 × 3, 4 × 4 × 4, and 5 × 5 × 5 *q*-point grids with 6, 8, 21, and 27 unique *q*-points, respectively, and performed the calculations at 10, 20, 30, 40, 50, 60, and 300 K. The *ab initio* line widths vary both as a function of wavevector and mode energy (Figure 3a), but we find that their behavior is relatively insensitive to the choice of grid (Figure S2). There is also a marked temperature dependence (Figures S3 and S4), arising from larger scattering probabilities as the phonons are more heavily populated at elevated temperature,^44^ which above 30 K is well approximated as Γ ∝ *T*, in agreement with the high-temperature limit of eq 1.^25^ However, the line widths predicted by eq 1 differ substantially from our *ab initio* calculated values, especially at low and high phonon energies (Figure 3a). The *ab initio* phonon line widths for **1** at 300 K are on the order of 0.1–40 cm^–1^ (corresponding to lifetimes on the order of 100–0.1 ps), and some modes become much longer lived at low temperatures with lifetimes of up to 4.5 ns (line widths of 0.001 cm^–1^) at 10 K. However, even the longest of these *ab initio* lifetimes is still orders of magnitude shorter than the experimental spin lifetimes for this compound, which are seconds to hundreds of microseconds, justifying the commonly assumed Born–Markov approximation for molecular spin dynamics.^22,31^ To the best of our knowledge, the present study represents one of very few explicit calculations of the phonon line widths and lifetimes for a molecular crystal and the only such calculation for a molecular magnet.

A central quantity of interest for molecular spin dynamics is the phonon DoS. The DoS is often obtained evaluating phonon frequencies on a chosen *q*-point grid and applying Gaussian or Lorentzian smoothing functions with an arbitrary line width (e.g., Figure 2b). Here, because we have calculated the mode-dependent line widths from first principles, we can directly construct the DoS without an artificial smoothing function (Figure 3b). The resulting low-energy DoS is sharply featured when using a small 2 × 2 × 2 *q*-point grid due to the coarse sampling of the Brillouin zone. The low-energy DoS as ω → 0, determined solely by the three acoustic modes, is expected to be a quadratic function of frequency,^64^ and this behavior in the low-energy DoS is better approximated using a larger 5 × 5 × 5 *q*-point grid to sample the dispersion of these modes more accurately, although we note that this grid still does not fully capture the low-energy dispersion (cf. Figure 2b). We also note that using a small grid would be more problematic at lower temperatures given the drastic narrowing of the line widths (Figure S5). Given the similar profiles of the line widths obtained with different grids (Figure S2), the difference in the DoS obtained with the different *q*-point sampling in Figure 3b can be attributed to better integration of the reciprocal space. We note that the issue of a sharply featured DoS can potentially be avoided by using a suitable fixed phonon line width, and a line width of Γ = 10 cm^–1^ gives a smooth DoS even with a comparatively sparse 2 × 2 × 2 *q*-point grid (Figure S6).

### Spin Dynamics and Crystalline Electrostatic Potential

To examine the influence of *ab initio* phonon line widths on the spin dynamics, we proceeded to calculate the spin–phonon coupling and magnetic relaxation rates (see the Methods). We first examined the spin dynamics under the gas-phase ansatz by optimizing the geometry and obtaining the vibrational modes of an isolated molecule of **1** (see the Methods). The calculated single-phonon Orbach magnetic relaxation rates, using the gas-phase vibrational modes and fixed line widths, show excellent agreement with the experimental measurements at high temperature (Figure S7). As we have shown previously, the absolute rates show a significant dependence on the choice of line width,^15,22,65^ with broader line widths leading to faster relaxation rates. This behavior occurs for single-phonon processes because there is only one possible phonon energy ℏω = |*E*_f_ – *E*_i_| that can cause a spin transition between any pair of states i and f (where *E*_i_ and *E*_f_ are the initial and final electronic state energies, respectively), and a larger line width gives a larger probability of overlap between |*E*_f_ – *E*_i_| and the calculated vibrational energies. We find that the best agreement is obtained with Γ = 1 cm^–1^ (Figure S7), which is consistent with the approximate center of the distribution of *ab initio* phonon line widths at 50 K (Figure 3a).

**Figure 4:**
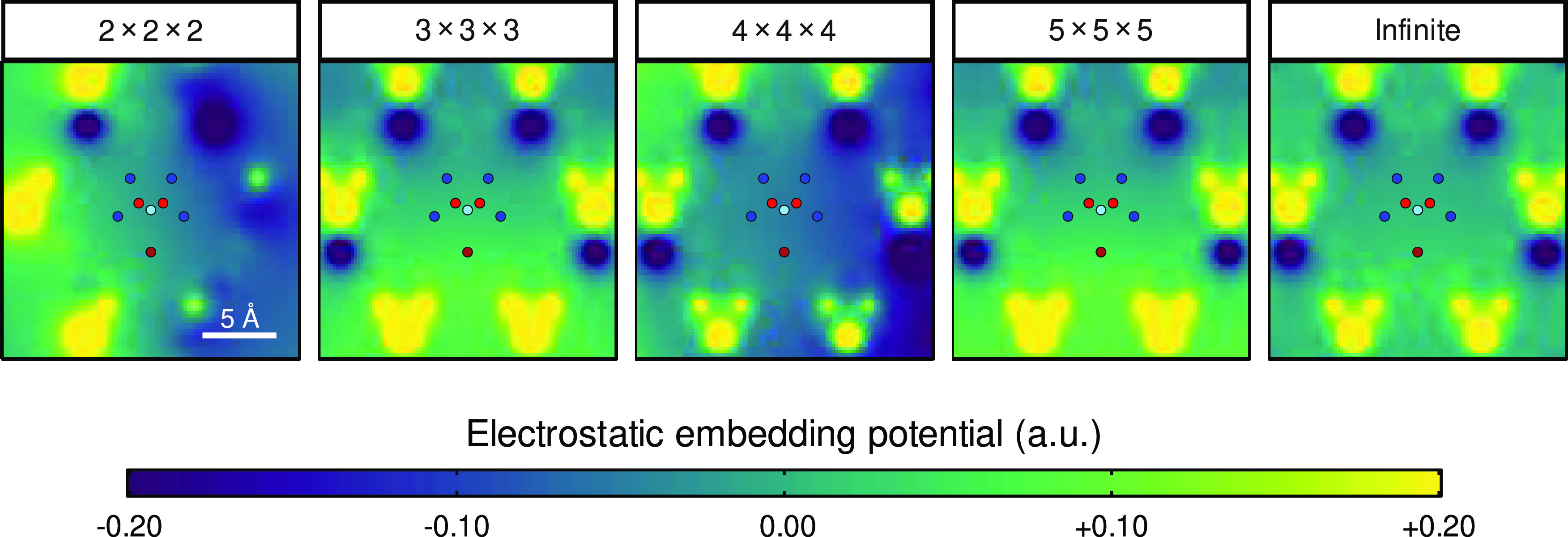
Electrostatic potential map in the vicinity of the central molecule of **1** for various finite slab expansions compared to the true infinite crystalline electrostatic potential. The positions of the Dy in the central molecule and the O, N, and Br atoms in the first coordination sphere are shown by circles colored as follows: Dy = light blue, O = red, N = blue, and Br = brown.

Two-phonon Raman rates can only be reliably obtained by considering the (pseudo)acoustic phonons in the solid-state, so this calculation cannot be performed using the gas-phase vibrational spectrum. We therefore also calculated the spin–phonon coupling and magnetic relaxation rates using the solid-state phonon modes and finite slab expansions commensurate with the *q*-point grids on which the *ab initio* frequencies and line widths were obtained. However, it is known that the crystalline electrostatic potential converges very slowly in real space, and finite slab expansions of lattice charges do not correctly approach the exact potential.^66^ This is because atomic charges within a unit cell are only balanced by their periodic neighboring charges, and so the surfaces of a finite slab remain charged, generating a static electric field that deviates from that in the infinite crystal.^66^ This is clearly observed in the electrostatic potential map, where there is an obvious lack of symmetry for the finite slab expansions compared to the exact result for an infinite crystal (Figure 4) and also in the corresponding equilibrium electronic CF splitting for **1** calculated with different electrostatic potentials (Figure S8). We note that we checked larger slabs up to a 9 × 9 × 9 expansion and still did not observe convergence (Figure S8). The electrostatic potential can be converged very quickly in reciprocal space using Ewald summation,^67^ but this method is not available in OpenMolcas.^53^ Luckily, there are two methods that can be employed to address this issue: the first uses an extra set of charges external to the crystalline slab as fitting parameters to replicate the exact infinite crystalline potential^68,69^ and the second places an approximately spherical array of unit cells in a perfect conductor to screen the charges directly,^70^ thus approximating the infinite crystalline potential very closely. The first method is not compatible with our analytic “one-shot” LVC method for obtaining the spin–phonon coupling coefficients,^60^ and we have therefore used the conductor screening method, which allows us to obtain spin–phonon coupling coefficients corrected for the infinite crystalline electrostatic potential (see the Methods).

To illustrate the effect that the finite slab expansions and thus inaccurate electrostatic potentials have on the spin dynamics, we have computed single-phonon (Orbach) and two-phonon (Raman) rates using fixed phonon line widths for four finite slab expansions (approach 1, see the Methods). We observe that the rates calculated using finite slab expansions oscillate around the experimental rates for even and odd slabs and do not converge (Figure S9). Using even and odd slab expansions yields faster and slower rates, respectively, which is consistent with smaller (larger) CF splitting for the former (latter). While the nonconvergence of the infinite electrostatic potential is indeed well known,^66^ we were surprised to see the large impact that a small electric potential developed over a finite slab can have on the spin dynamics of molecular crystals, affecting the magnetic relaxation rates by an order of magnitude. Indeed, it is not the spin–phonon coupling itself that is affected by this approximation,^60^ but rather the static electronic structure of the molecule, and this directly leads to the observed effect. We therefore adopt a method that allows us to include the infinite crystalline electrostatic potential (Approach 2, see the Methods), but we still compare different *q*-point grids to assess the impact of Brillouin zone integration on the phonons and spin dynamics.

With this improved approach, calculation of the single-phonon rates in the Orbach regime with fixed phonon line widths shows excellent agreement with the experimental rates (Figure S10), and as for the gas-phase calculation, the rates are positively correlated with the choice of the line width. However, the dependence on line width is far less significant than in the gas phase and decreases when using larger *q*-point grids: the rates obtained with Γ = 0.1 and 10 cm^–1^ differ by a factor of ∼19 at 60 K in the gas-phase calculations, reducing to factors of ∼8 and ∼2 for solid-state calculations with 2 × 2 × 2 and 5 × 5 × 5 grids, respectively. We next calculated the two-phonon Raman rates with fixed line widths, which are in excellent agreement with the experimental rates (Figure S11). We note that the choice of line width has a larger effect in this region than in the single-phonon Orbach region and that, counterintuitively, the two-phonon Raman rates have a negative correlation with line width, i.e., the rates become slower with larger line widths, which is the opposite behavior to the single-phonon rates. This can be explained by the fact that for the Raman mechanism, only the difference in the phonon energy ℏω–ℏω′ must match the difference in electronic energy *E*_f_ – *E*_i_, which allows many pairs of phonons to cause a transition between the ground doublet states.^45^ When the line widths are larger, the phonon lineshapes extend to higher energies, resulting in smaller Bose–Einstein occupation factors and hence a reduction in the magnitude of these contributions.

As for the single-phonon Orbach rates, we observe that the dependence on the fixed line width becomes smaller with improved reciprocal-space integration (the rates for Γ = 0.1 and 10 cm^–1^ differ by a factor of 1.5 at 60 K for the 2 × 2 × 2 grid, which reduces to 1.2 for the 5 × 5 × 5 grid). We also note that the increased sensitivity of the magnetic relaxation rates in the Raman region to the choice of phonon line width compared to the Orbach region could provide an explanation for the increase in the distribution of magnetic relaxation rates for this compound as the temperature is decreased, i.e., the presence of crystalline disorder, shown to correlate with the width of the distributions of magnetic relaxation rates,^71^ has more of an effect on the Raman rates, which tend to dominate as the temperature is reduced.

We now examine the impact of using the *ab initio* phonon line widths. In the Orbach region, the rates obtained using mode- and temperature-dependent line widths coincide with the fixed line width calculations using Γ = 1 cm^–1^ (Figure S10). In the Raman region, the rates obtained with mode- and temperature-dependent line widths are close to those obtained with fixed Γ = 10 cm^–1^ at 60 K but increasingly approach the smaller fixed line width calculations at lower temperature, crossing the Γ = 0.1 cm^–1^ rates between 20 and 10 K (Figure S11). As the Raman rates are more strongly affected by the choice of line width than the Orbach rates, it is unsurprising that the extreme narrowing of some of the *ab initio* phonon line widths at low temperatures has a marked impact on the relaxation rates. However, we note that the profile of the relaxation rates calculated using *ab initio* mode- and temperature-dependent line widths does not agree with the experimental data at the lowest temperatures and in particular level off at low temperature, while the experimental rates continue to decrease. We therefore suggest that the extreme narrowing of the *ab initio* line widths at low temperatures is overestimated and that there are likely other sources of phonon scattering (such as boundary effects, impurities, defects, and/or disorder) in real crystals that would lead to shorter phonon lifetimes, and therefore broader line widths, at lower temperature than those estimated by our DFT calculations on a perfect infinite crystal.

**Figure 5:**
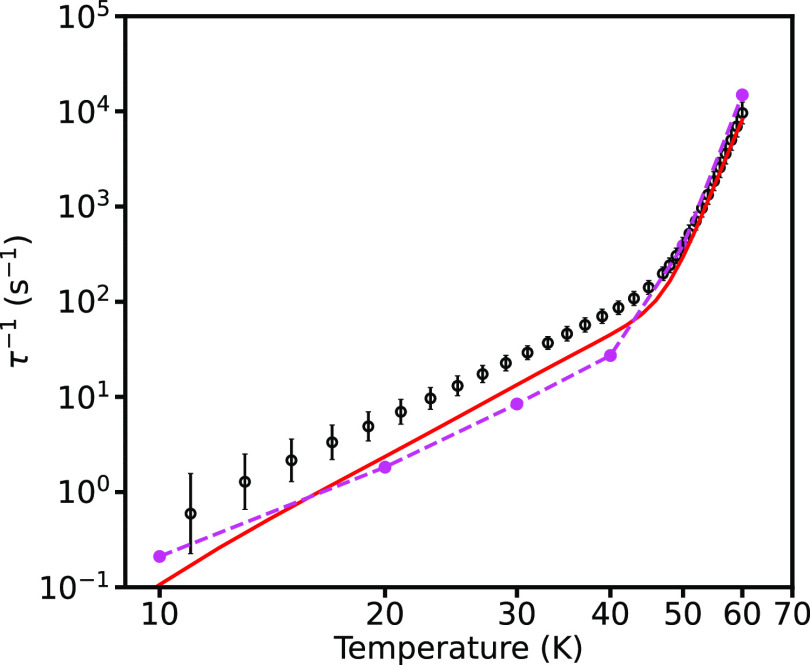
Experimental (black circles) and calculated magnetic relaxation rates for **1**. Calculations are performed using the solid-state phonon modes on the 5 × 5 × 5 *q*-point grid, including the infinite crystalline electrostatic potential, and considering both single-phonon and two-phonon transitions, with a fixed Γ = 0.1 cm^–1^ (red) and *ab initio* mode- and temperature-dependent line widths (pink points and dashed lines, respectively). The error bars on the experimental data points denote one estimated standard deviation of the distribution of relaxation rates.^71^

Finally, we compare our results to the effective NVT phonon line width proposed by Lunghi et al. (eq 1). During our calculations, we found that the NVT line widths predicted for the low-energy phonons increase drastically at high temperature (e.g., for ℏω = 1 cm^–1^, Γ = 417 cm^–1^ at 300 K from eq 1, but is ca. 1–10 cm^–1^ as calculated *ab initio*, Figures S3 and S4) such that the numerical integration over the phonon lineshapes when computing the Raman mechanism becomes problematic, and we therefore only report these results for *T* ≤ 46 K. We have also previously shown that the NVT line widths of the high-energy phonons drastically narrow at low temperature, resulting in unphysical Orbach rates,^22^ which again we find here when using anti-Lorentzian lineshapes. Nevertheless, we find that in both the Orbach and Raman regions, the NVT expression gives rates that are close to those obtained using a fixed Γ = 10 cm^–1^ (Figures S12 and S13) and in fact gives rates nearly identical to those obtained by fixing the line widths to their *ab initio* calculated values at 300 K, which themselves agree well with the rates obtained using the mode- and temperature-dependent line widths above ca. 30 K (Figures S12 and S13). Overall, however, excellent agreement with the experimental rates can be obtained using a simple fixed line width of Γ = 0.1 cm^–1^ (Figure 5), but, there is little difference to using Γ = 1 cm^–1^ when the largest 5 × 5 × 5 *q*-point grid is employed (Figure S14).

While it is well known that high-energy localized molecular vibrations drive magnetic relaxation in the Orbach regime,^15,16^ it is not very well understood which phonon modes drive magnetic relaxation in the Raman regime—this is due to both the inability of experiments to probe these two-phonon interactions directly and also the convoluted nature of the two-phonon process itself.^45^ Given the excellent reproduction of the experimental data using our *ab initio* methodology, we are in the unique position to reliably decompose our calculations and learn which phonon modes are important for the Raman mechanism in compound **1**. As the Raman rates are calculated as sums over pairs of modes involved in the scattering process (eqs 46–49 in reference^45^), we can examine the contribution that every pair makes to the total rate; here, we do so using the modes obtained with the 2 × 2 × 2 *q*-point grid with fixed Γ = 1 cm^–1^ for simplicity.

At 10 K, Raman relaxation is dominated by scattering between four modes (Table S2): between ℏω = 19.07 cm^–1^ at ***q*** = *Y* (0.5, −0.5, 0) and one of the doubly degenerate modes with ℏ*ω* = 19.98 cm^–1^ at ***q*** = *T* (0.5, −0.5, 0.5), and between one of the doubly degenerate modes with ℏ*ω* = 27.41 cm^–1^ at ***q*** = (0.5, 0, 0.5) and ℏω = 27.66 cm^–1^ at ***q*** = *Y* (0.5, −0.5, 0). These are all off-Γ acoustic modes that mix substantially with the low-energy pseudoacoustic spectrum (Figure 2a). At higher temperatures where the Raman mechanism is almost overtaken by the Orbach mechanism (e.g., 40 K; Table S3), significant contributions arise from scattering between higher energy modes. One pair has ℏ*ω* = 154.68 cm^–1^ (mode 42 at ***q*** = *Y* (0.5, −0.5, 0), corresponding to an asymmetric N_py_–Dy–N_py_ stretch) and ℏω = 154.74 cm^–1^ (degenerate modes 43 and 44 at ***q*** = *Z*, corresponding to a pinching of the phenoxide O atoms parallel to the Dy–Br axis accompanied by a rotation of the O atoms around the Dy–Br axis). A second pair has ℏω = 113.65 cm^–1^ (degenerate modes 29 and 30 at ***q*** = *T* (0.5, −0.5, 0.5), a twisting of the phenoxide and pyridine rings around their tethers) and ℏω = 114.47 cm^–1^ (mode 30 at ***q*** = (0.5, 0, 0), which is a pinching of the pyridyl N and phenoxide O atoms parallel to the Dy–Br axis).

While acoustic modes are fundamental phenomena in the structural dynamics of solids and cannot be engineered to “turn off”, the Raman relaxation enabled by the higher energy optical modes can in theory be tuned with chemistry. However, we find that artificially excluding these six higher energy modes and their symmetry equivalents (ℏω = 154.68, 154.74, 113.65, and 114.47 cm^–1^) from our Raman relaxation rate calculations leads to a barely perceptible change in the total rate of only <11% (Table S4). Removing all modes listed in Table S3 and their symmetry equivalents leads to a slightly more substantial reduction of 27% at 40 K, but it is not until we additionally remove the pseudoacoustic modes between 25 and 60 cm^–1^ that contribute to the first peak in the phonon DoS (Figure 2b) that we obtain a 66% average reduction in the Raman rates across the 10–40 K region (Table S4). The pseudoacoustic modes involve molecular rotations and twists, some of which are accompanied by wagging of the Dy–Br bond, which also occurs at low energies given the large mass of Br. The terminal bromide ligand can therefore be associated with increasing Raman relaxation rates in **1**, along with the flexible backbone of the bbpen ligand, as described above.

These findings again advocate for rigid, multihapto ligands that avoid single-donor atom coordination groups in order to suppress the Raman relaxation mechanism.^20^ Indeed, these guidelines are in agreement with those proposed by Sessoli, Lunghi, and co-workers, who suggested that to reduce Raman relaxation, one ought to (i) “have a small amount of low-energy vibrations in both the lattice and the molecular unit” and (ii) “use rigid ligands able to decouple intramolecular motions from low-energy acoustic vibrations”;^31^ our updated guidance gives some further context on how this can be achieved, by avoiding single-donor atom ligands and terminal halides.

## Conclusions

In this work, we have presented our mature methodology for the calculation of molecular spin–phonon coupling in the solid state. Using a case study of a Dy(III) single-molecule magnet, we have shown that significant errors arise when employing finite crystalline slabs but that these can be corrected by employing a perfect conductor screening model, which is compatible with our accurate and efficient calculation of spin–phonon coupling constants. We have also performed an *ab initio* calculation of the phonon lifetimes and line widths and demonstrated that (i) the Born–Markov approximation is justified for Dy(III) single-molecule magnets, and (ii) that the choice of the phonon line width model is not crucial to describe molecular spin dynamics, given an adequate integration of reciprocal space. Hence, the large computational burden of obtaining *ab initio* line widths is not justified for this application, and either the NVT approximation or a simple fixed line width on the order of Γ = 0.1–10 cm^–1^ is the most transferrable and economic method for molecular spin dynamics calculations. Indeed, we are currently exploring the present method applied to other molecular magnets, including those based on different metal ions, and find excellent agreement with experiment. The open-source tools we have developed and used herein thus open the door to quantitatively accurate and efficient spin–phonon calculations on molecular solids.

## Supporting Information Available

Convergence testing, optimized unit cell parameters, *ab initio* calculated line widths, DoS plots, magnetic relaxation rates under different approximations, and contributions to Raman relaxation (PDF)
