# Supplementary material for: Accurate and Efficient Spin–Phonon Coupling and Spin Dynamics Calculations for Molecular Solids
Source: J Am Chem Soc. 2023 Nov 2;145(45):24558–67. doi: 10.1021/jacs.3c06015 (PMC10655086; doi:10.1021/jacs.3c06015)
Supplement: Supplementary file 1 — ja3c06015_si_001.pdf [file ja3c06015_si_001.pdf]

Supporting Information for Accurate and efficient spin-phonon coupling and spin dynamics calculations for molecular solids

Rizwan Nabi,[a,†] Jakob K. Staab,[a,†] Andrea Mattioni,[a] Jon G. C. Kragskow,[a,b] Daniel Reta,[a,c,d,e] Jonathan M. Skelton[a,*] and Nicholas F. Chilton[a,*]

[a] Department of Chemistry, University of Manchester, Manchester M13 9PL, UK

[b] Department of Chemistry, University of Bath, Bath, BA2 7AY, UK

[c] Faculty of Chemistry, University of the Basque Country UPV/EHU, 20018, Donostia, Spain

[d] Donostia International Physics Center (DIPC), 20018, Donostia, Spain

[e] IKERBASQUE, Basque Foundation for Science, 48013, Bilbao, Spain

[†] These authors contributed equally to this work.

Email: jonathan.skelton@manchester.ac.uk, nicholas.chilton@manchester.ac.uk

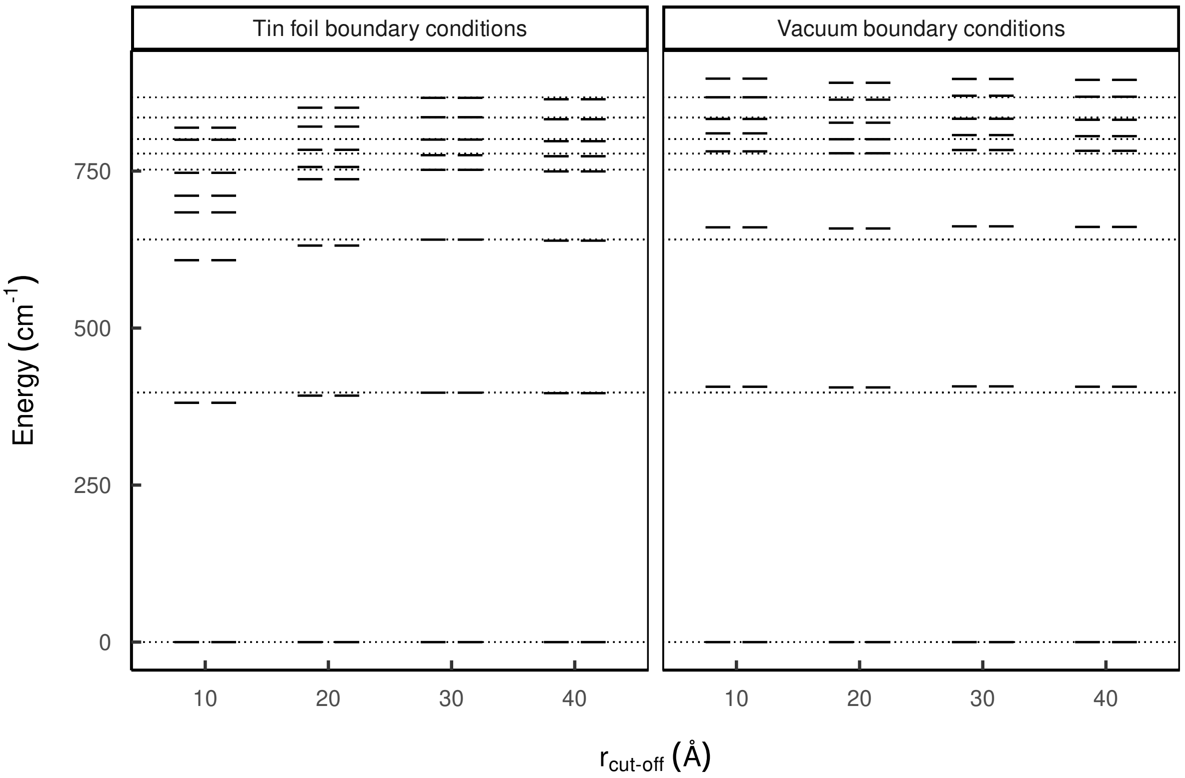

Figure S1. Convergence of the equilibrium CF energy levels of **1** using various "spherical" cut-off radii when embedded in a conductor reaction field (upper), compared to the same "spherical" clusters of unit cells without the conductor reaction field (lower).

Table S1. Measured and DFT-optimised primitive unit-cell parameters for **1**.

**Parameter**	**Experimental**[1]	**Optimised**
**a (Å)**	9.72377	9.62628
**b (Å)**	9.72377	9.62628
**c (Å)**	17.08300	17.01866
α **(°)**	90.0	90.0
β **(°)**	90.0	90.0
γ **(°)**	126.7619	126.7677

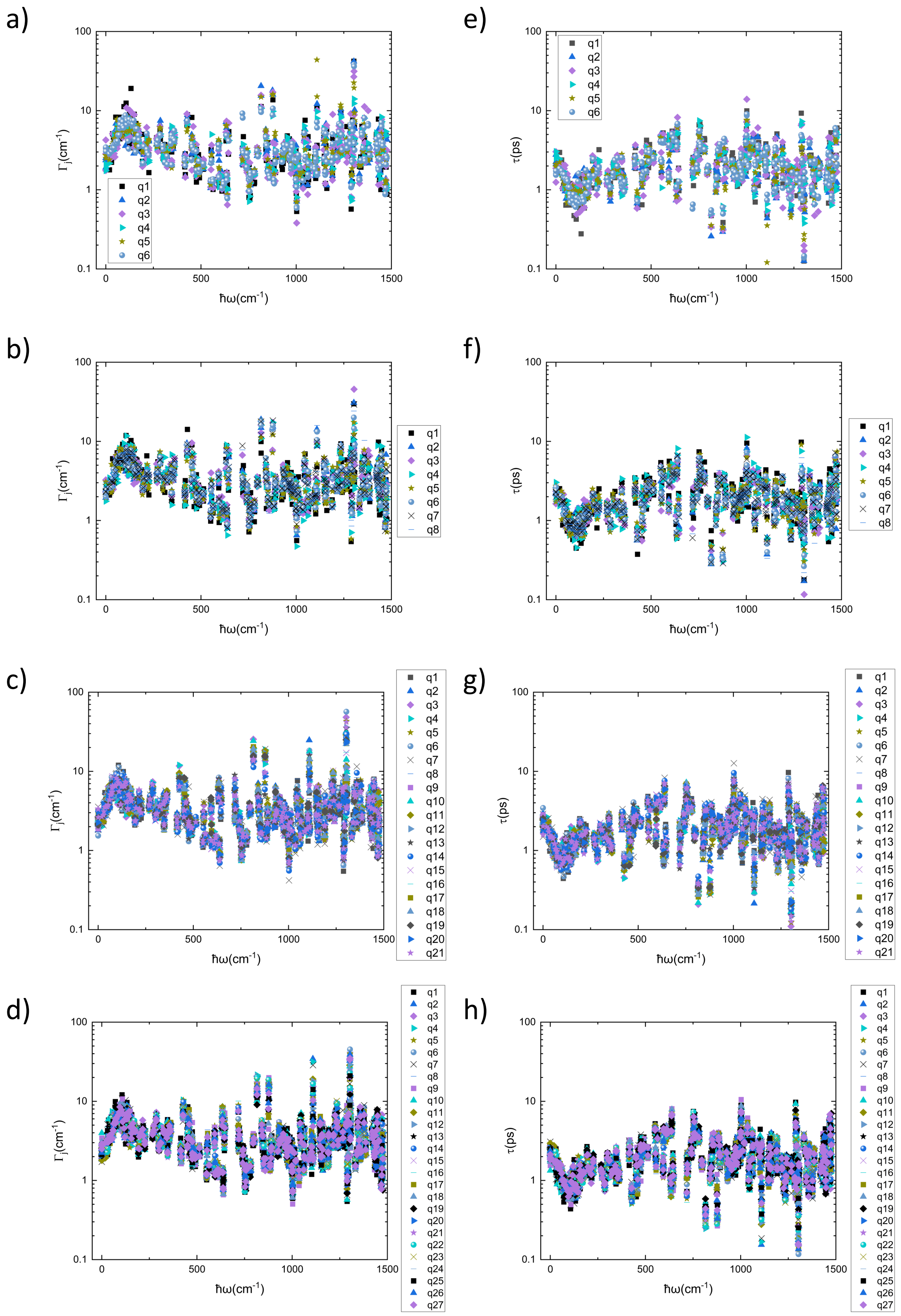

Figure S2. Calculated phonon linewidths (a-d) and lifetimes (e-h) at 300 K at each of the unique *q*-points in the 2×2×2 (a,e), 3×3×3 (b,f), 4×4×4 (c, g) and 5×5×5 (d, h) *q*-point grids, as a function of mode energy, in the low-energy region of the phonon spectrum.

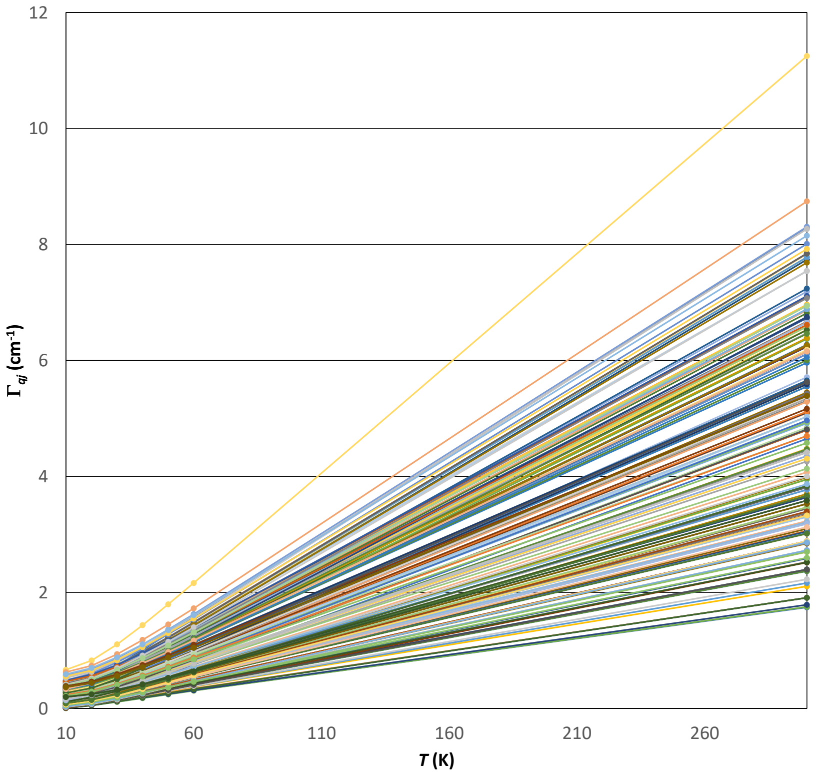

Figure S3. Calculated phonon linewidths for **1** as a function of temperature from 10-300 K for the 203 modes below 100 cm^{-1} at the 6 unique q-points on a 2×2×2 q-point grid. Points are connected by lines.

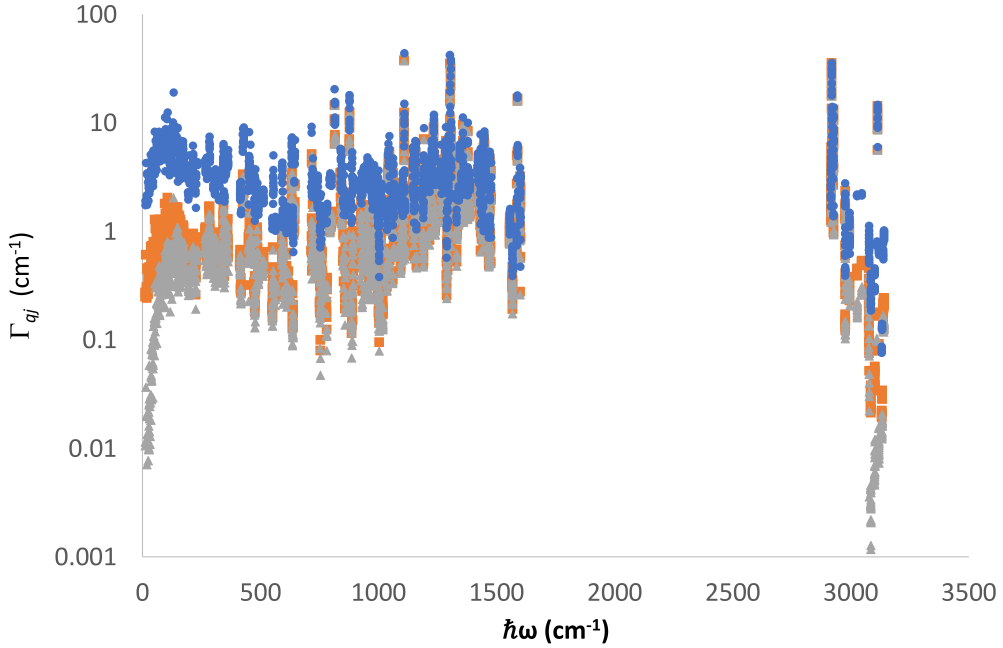

Figure S4. Calculated phonon linewidths for **1** as a function of mode energy obtained using a 2×2×2 q-point grid at 10 (grey), 50 (orange) and 300 K (blue).

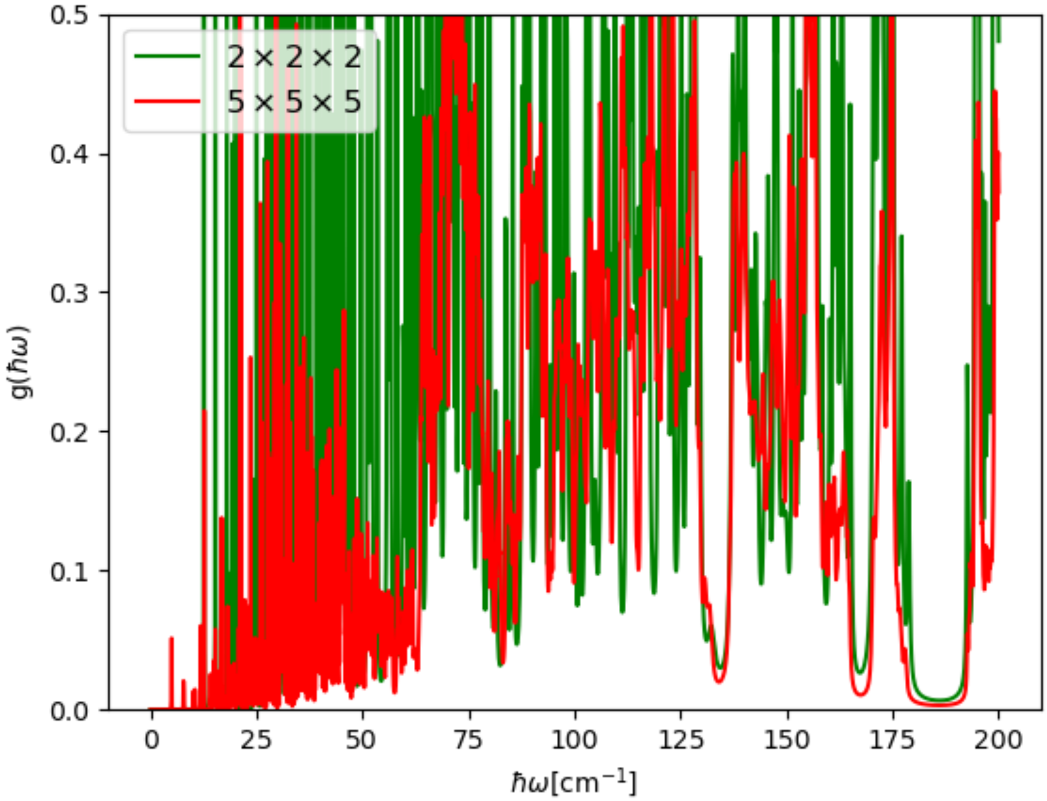

Figure S5. Low-energy DoS generated using the DFT-calculated mode energies and linewidths at 10 K obtained on 2×2×2 and 5×5×5 *q*-point grids, using an anti-Lorentzian lineshape. The y-axis shown on the same scale as Figure 3b in the main text.

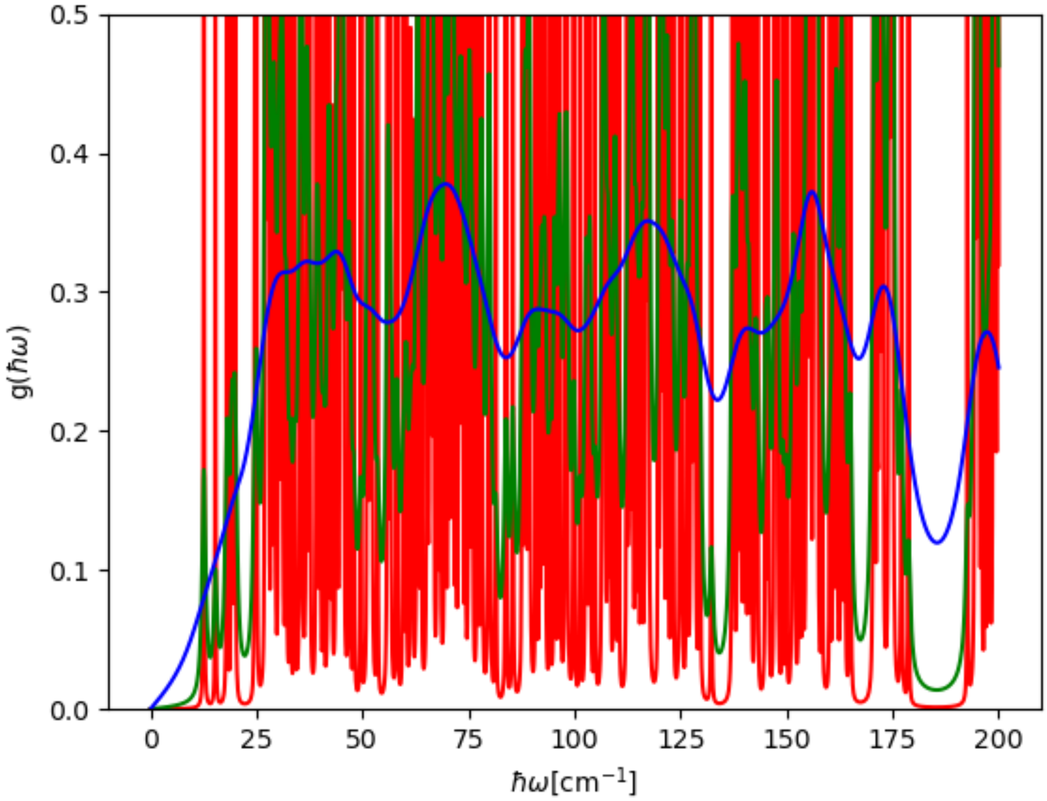

Figure S6. Low-energy DoS generated using the DFT-calculated mode energies using a 2×2×2 *q*-point grid and using fixed linewidths of 0.1 (red), 1 (green) and 10 cm^{-1} (blue), using an anti-Lorentzian lineshape. The y-axis shown on the same scale as Figure 3b in the main text.

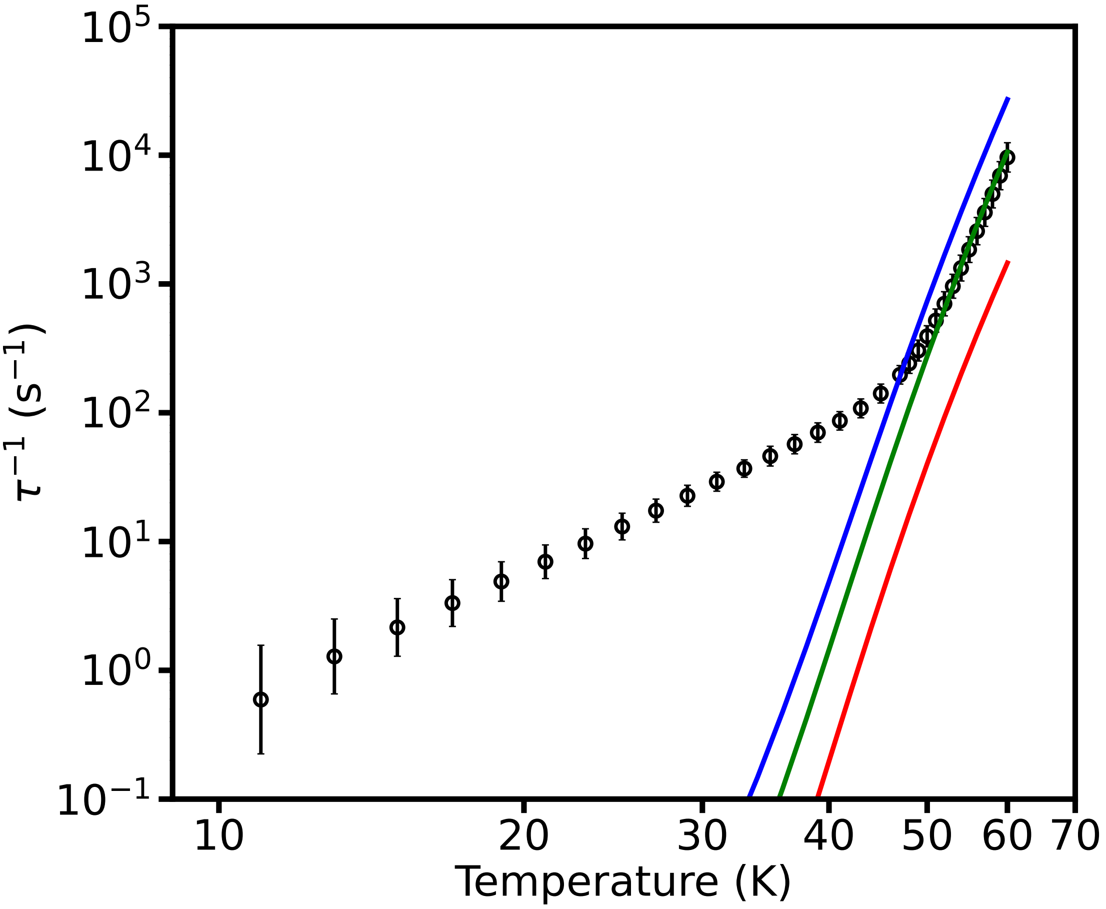

Figure S7. Experimental (black circles) and calculated magnetic relaxation rates for **1**. Calculations are performed using the gas-phase ansatz considering single-phonon transitions only, with fixed linewidths of Γ = 0.1 (red), 1 (green) and 10 (blue) cm^{-1}. The bars on the experimental data points denote one estimated standard deviation of the distribution of relaxation rates.[2]

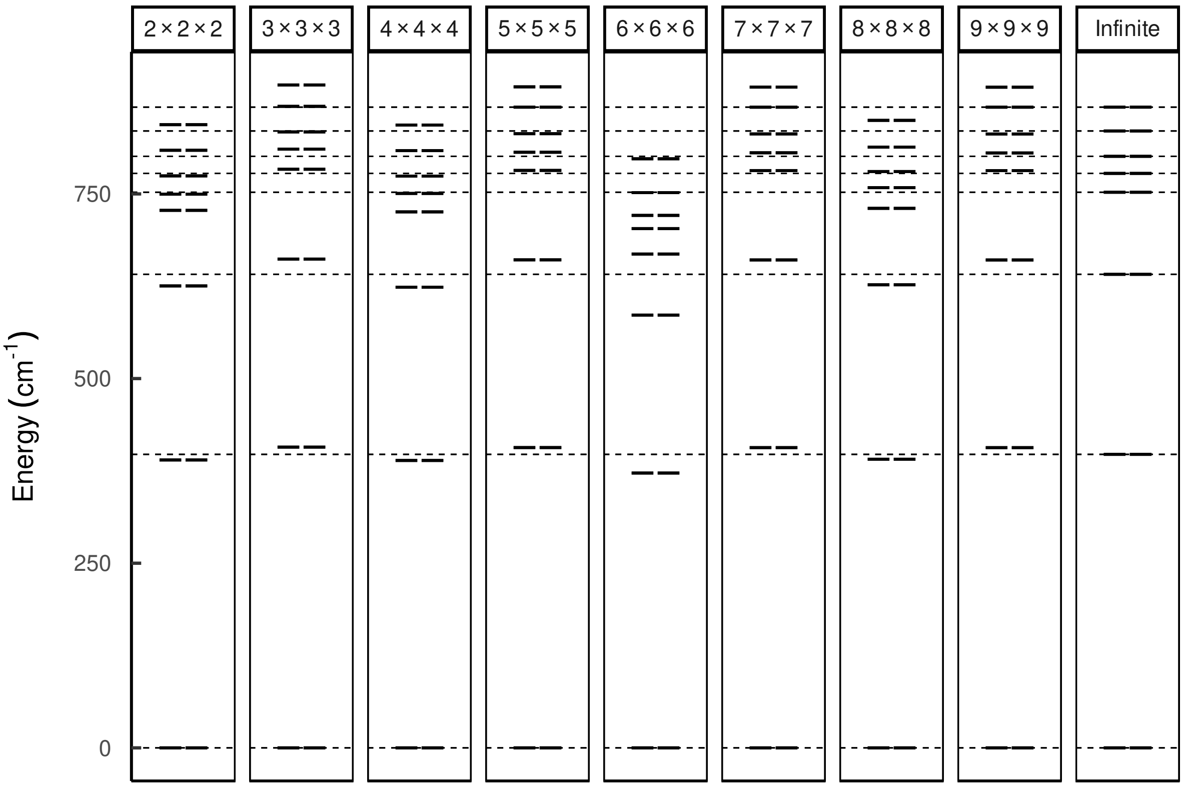

Figure S8. Equilibrium CF energy levels of **1** using various supercell expansions for determining the electrostatic potential, compared to those obtained using the true infinite crystalline electrostatic potential.

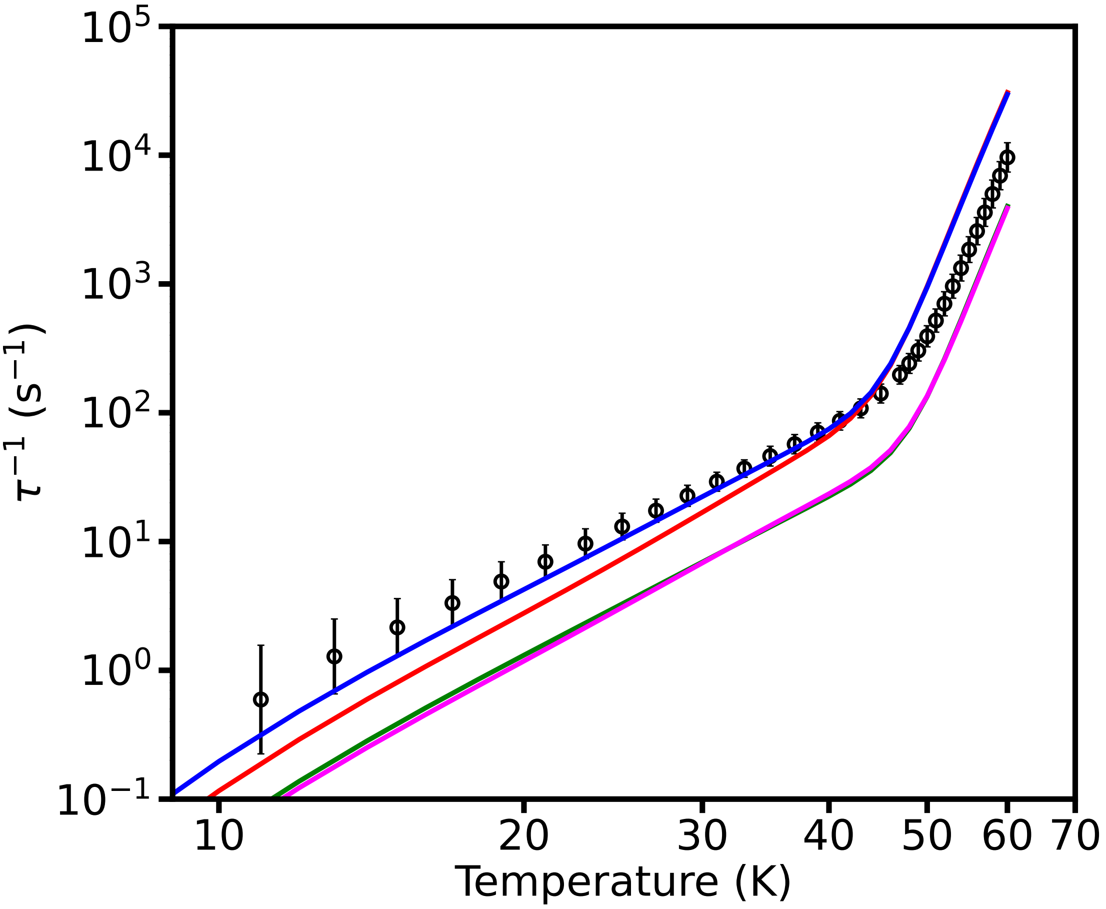

Figure S9. Experimental (black circles) and calculated magnetic relaxation rates for **1**. Calculations are performed using solid-state phonon modes on 2×2×2 (red), 3×3×3 (green), 4×4×4 (blue) and 5×5×5 (pink) *q*-point grids, for the same finite supercell expansions, considering single-phonon and two-phonon transitions, with a fixed linewidth of Γ = 1 cm^{-1}. These calculations were performed without accounting for the infinite crystalline electrostatic potential. The bars on the experimental data points denote one estimated standard deviation of the distribution of relaxation rates.[2]

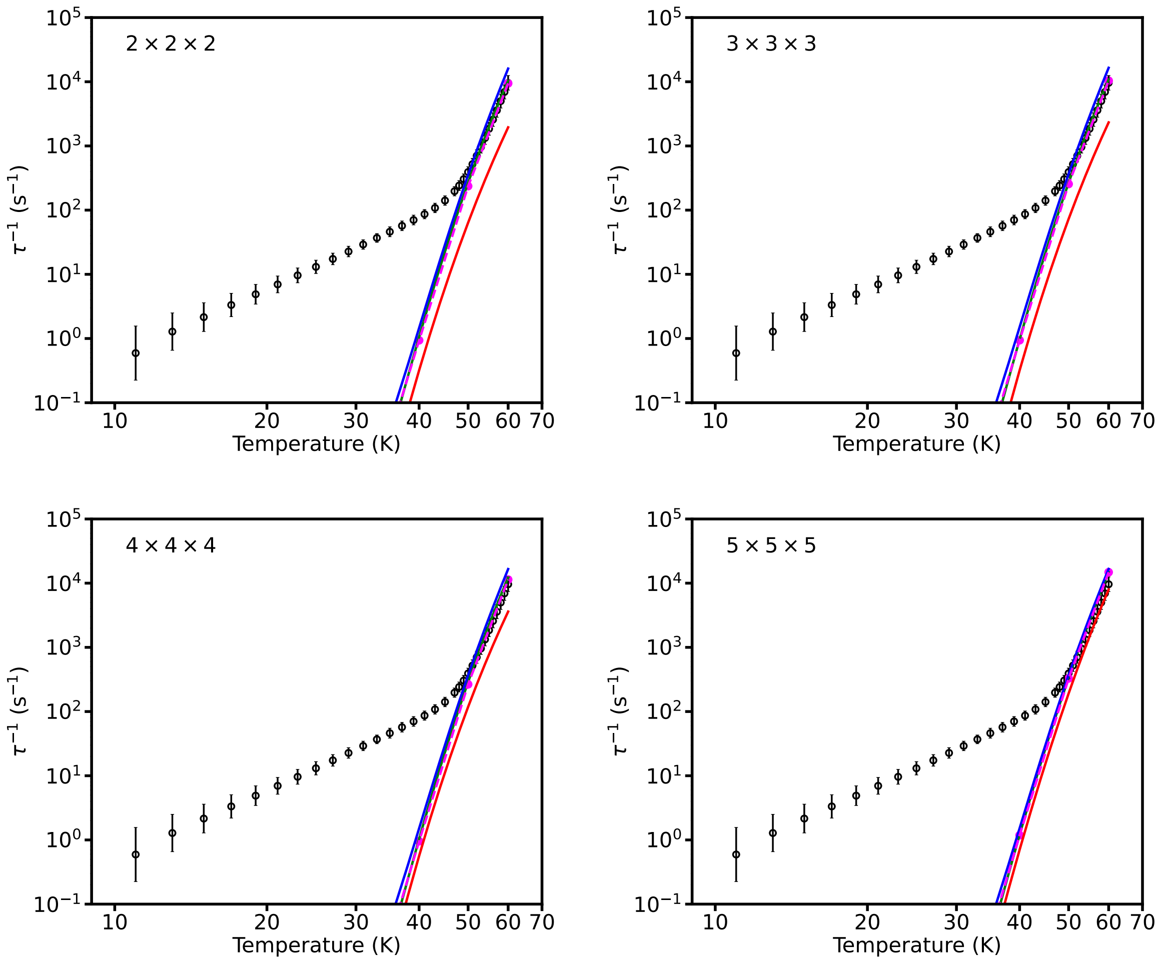

Figure S10. Experimental (black circles) and calculated magnetic relaxation rates for **1**. Calculations are performed using solid-state phonon modes on 2×2×2, 3×3×3, 4×4×4 and 5×5×5 *q*-point grids, including the infinite crystalline electrostatic potential, and considering single-phonon transitions only, with fixed linewidths of Γ = 0.1 (red), 1 (green) and 10 (blue) cm^{-1}, and *ab initio* (mode- and temperature-dependent) linewidths (pink points and dashed lines). The bars on the experimental data points denote one estimated standard deviation of the distribution of relaxation rates.[2]

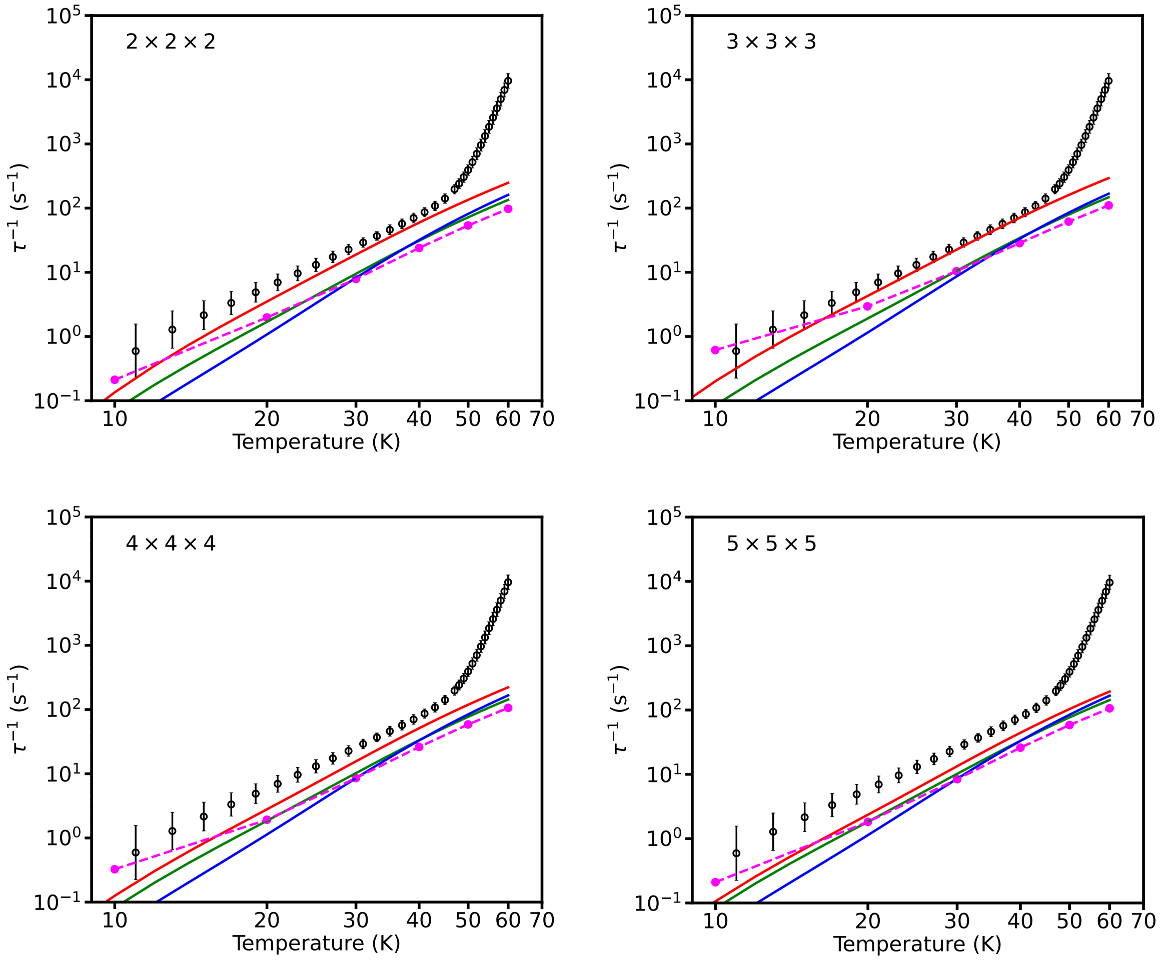

Figure S11. Experimental (black circles) and calculated magnetic relaxation rates for **1**. Calculations are performed using solid-state phonon modes on 2×2×2, 3×3×3, 4×4×4 and 5×5×5 *q*-point grids, including the infinite crystalline electrostatic potential, and considering two-phonon transitions only, with fixed linewidths of Γ = 0.1 (red), 1 (green) and 10 (blue) cm^{-1}, and *ab initio* (mode- and temperature-dependent) linewidths (pink points and dashed lines). The bars on the experimental data points denote one estimated standard deviation of the distribution of relaxation rates.[2]

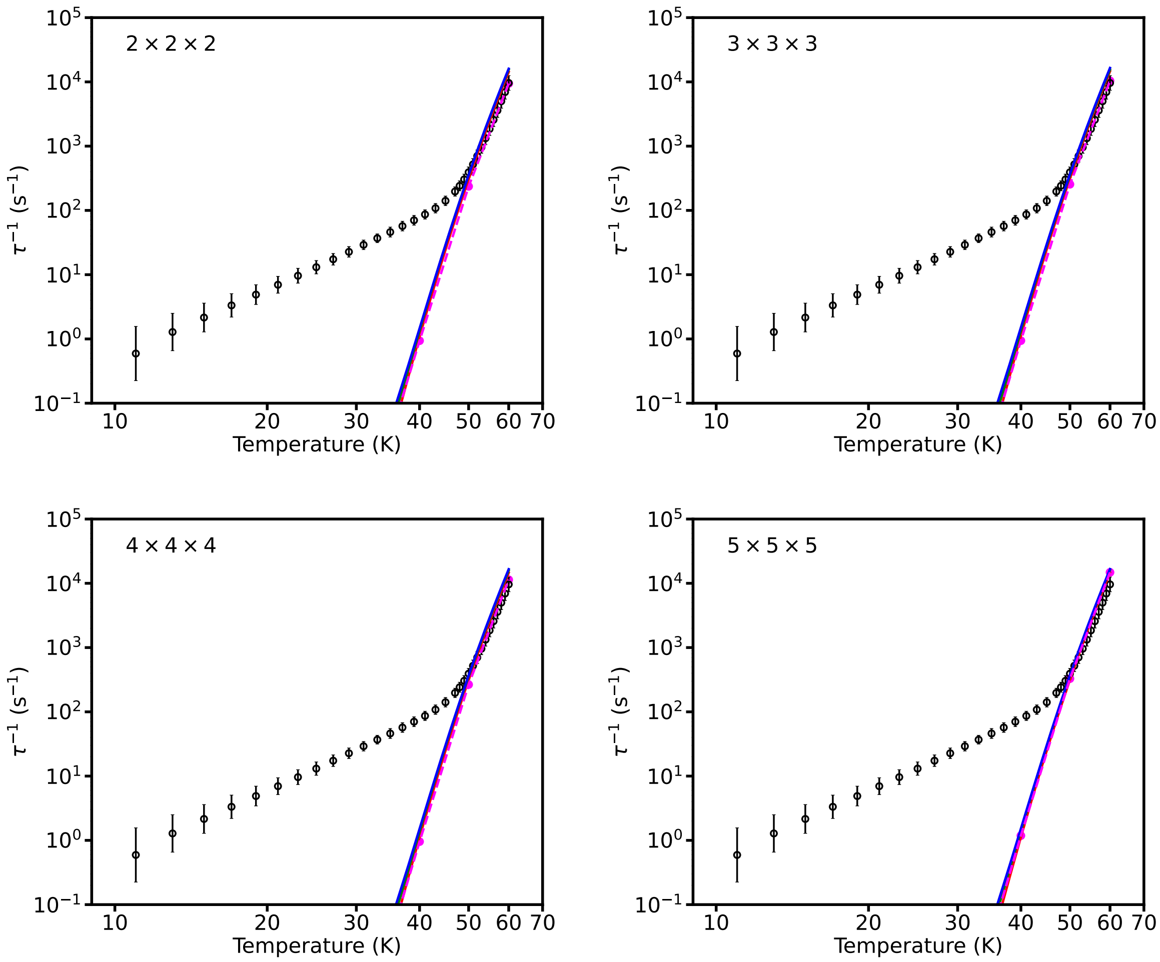

Figure S12. Experimental (black circles) and calculated magnetic relaxation rates for **1**. Calculations using solid-state phonon modes on 2×2×2, 3×3×3, 4×4×4 and 5×5×5 *q*-point grids, including the infinite crystalline electrostatic potential, and considering single-phonon transitions only. Linewidths given by Equation 1 in the main text (red), mode-dependent *ab initio* linewidths at 300 K (green), fixed Γ = 10 cm^{-1} (blue), and the *ab initio* (mode- and temperature-dependent) linewidths (pink points and dashed lines). The bars on the experimental data points denote one estimated standard deviation of the distribution of relaxation rates.[2]

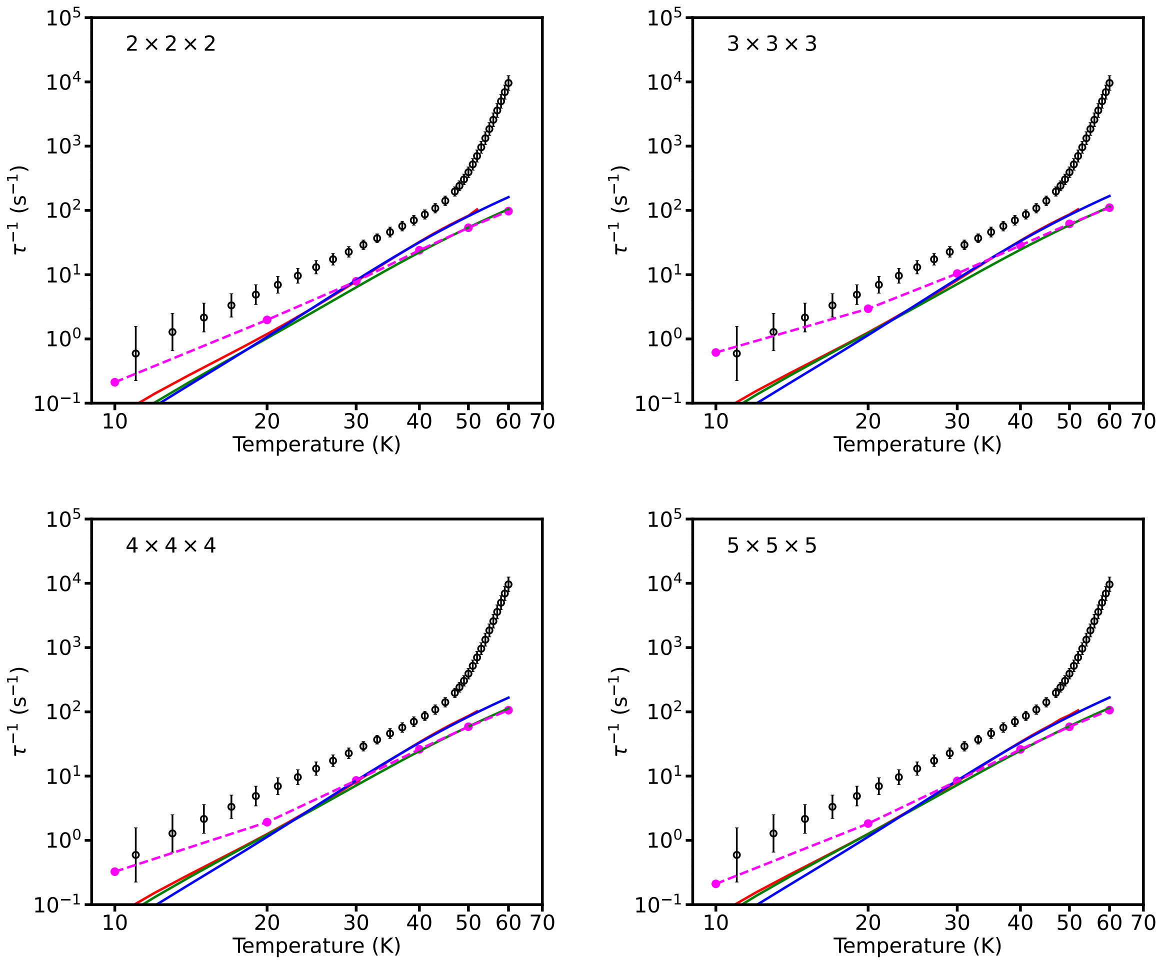

Figure S13. Experimental (black circles) and calculated magnetic relaxation rates for **1**. Calculations using solid-state phonon modes on 2×2×2, 3×3×3, 4×4×4 and 5×5×5 *q*-point grids, including the infinite crystalline electrostatic potential, and considering two-phonon transitions only. Linewidths given by Equation 1 in the main text (red), mode-dependent *ab initio* linewidths at 300 K (green), fixed Γ = 10 cm^{-1} (blue), and the *ab initio* (mode- and temperature-dependent) linewidths (pink points and dashed lines). The bars on the experimental data points denote one estimated standard deviation of the distribution of relaxation rates.[2]

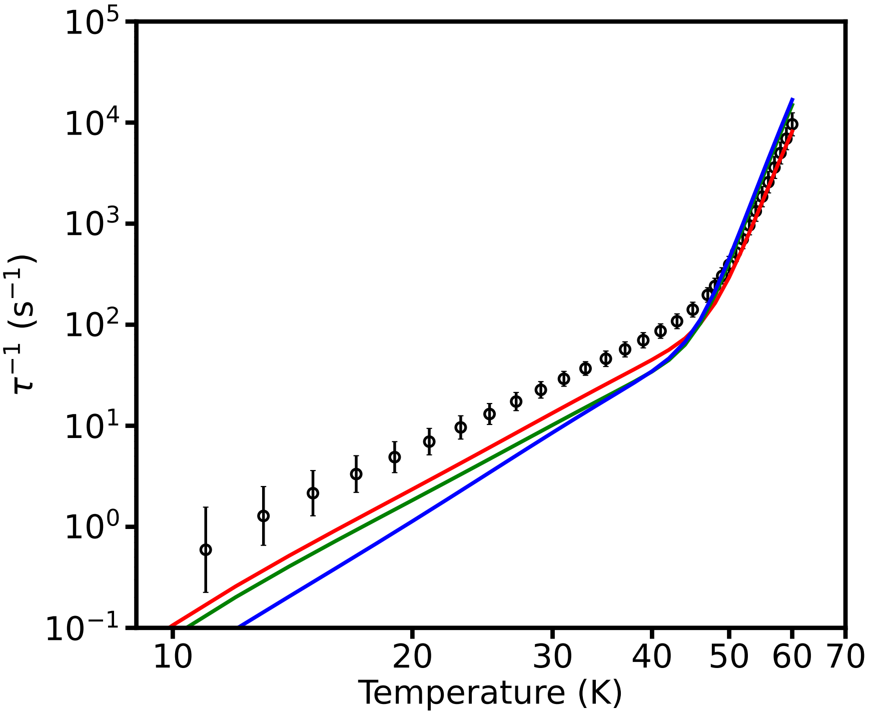

Figure S14. Experimental (black circles) and calculated magnetic relaxation rates for **1**. Calculations using solid-state phonon modes on a 5×5×5 *q*-point grid, including the infinite crystalline electrostatic potential, considering both single-phonon and two-phonon transitions, with fixed linewidths of Γ = 0.1 (red), 1 (green) and 10 (blue) cm^{-1}. The bars on the experimental data points denote one estimated standard deviation of the distribution of relaxation rates.[2]

Table S2. Contributions to Raman rates for **1** at 10 K, calculated using a 2×2×2 *q*-point grid with Γ = 1 cm^{-1}. Only relative contributions > 0.5 shown.

**Mode 1 (cm^{-1})**	**Mode 2 (cm^{-1})**	**Relative contribution**
19.07	19.98	1.00
27.41	27.66	0.88
35.40	36.82	0.68
27.41	27.85	0.67
30.23	30.49	0.54
30.23	30.49	0.53

Table S3. Contributions to Raman rates for **1** at 40 K, calculated using a 2×2×2 q-point grid with $\Gamma = 1\ cm^{-1}$. Only relative contributions > 0.5 shown.

Mode 1 (cm^{-1})	Mode 2 (cm^{-1})	Relative contribution
154.68	154.74	1.00
113.65	114.47	0.90
113.65	114.47	0.89
120.36	121.07	0.64
157.14	157.29	0.62
156.60	157.14	0.59
156.60	157.14	0.58
156.86	157.14	0.56
120.36	120.70	0.55
124.82	125.18	0.54
124.82	125.18	0.52
156.86	157.14	0.51

Table S4. Calculated relaxation rates with some modes removed. Column **A**: all modes included. Column **B**: six high energy modes ($\hbar\omega$ = 154.68, 154.74, 113.65 and 114.47 cm^{-1}) and their symmetry equivalents removed. Column **C**: all modes listed in Table S3 and their symmetry equivalents removed. Column **D**: all modes listed in Table S3 and their symmetry equivalents, as well as all modes between 25 and 60 cm^{-1} removed.

Temperature (K)	A (s^{-1})	B (s^{-1})	C (s^{-1})	D (s^{-1})	B/A	C/A	D/A
40	31.026	27.515	22.766	15.312	0.89	0.73	0.49
30	9.492	8.733	7.840	4.202	0.92	0.83	0.44
20	1.692	1.651	1.614	0.500	0.98	0.95	0.30
10	0.071	0.071	0.071	0.009	1.00	1.00	0.13

References

(1) Liu, J.; Chen, Y.-C.; Liu, J.-L.; Vieru, V.; Ungur, L.; Jia, J.-H.; Chibotaru, L. F.; Lan, Y.; Wernsdorfer, W.; Gao, S.; Chen, X.-M.; Tong, M.-L. A Stable Pentagonal Bipyramidal Dy(III) Single-Ion Magnet with a Record Magnetization Reversal Barrier over 1000 K. *J. Am. Chem. Soc.* **2016**, *138* (16), 5441–5450.

(2) Reta, D.; Chilton, N. F. Uncertainty Estimates for Magnetic Relaxation Times and Magnetic Relaxation Parameters. *Phys. Chem. Chem. Phys.* **2019**, *21* (42), 23567–23575.
